# Skeletal muscle myosin heavy chain fragmentation as a potential marker of protein degradation in response to resistance training and disuse atrophy

**DOI:** 10.1113/EP092093

**Published:** 2024-08-24

**Authors:** Daniel L. Plotkin, Madison L. Mattingly, Derick A. Anglin, J. Max Michel, Joshua S. Godwin, Mason C. McIntosh, Nicholas J. Kontos, João G. A. Bergamasco, Maíra C. Scarpelli, Vitor Angleri, Lemuel W. Taylor, Darryn S. Willoughby, C. Brooks Mobley, Andreas N. Kavazis, Carlos Ugrinowitsch, Cleiton A. Libardi, Michael D. Roberts

**Affiliations:** ^1^ School of Kinesiology Auburn University Auburn Alabama USA; ^2^ MUSCULAB – Laboratory of Neuromuscular Adaptations to Resistance Training, Department of Physical Education Federal University of São Carlos – UFSCar São Carlos SP Brazil; ^3^ School of Health Professions University of Mary Hardin‐Baylor Belton Texas USA; ^4^ School of Physical Education and Sport University of São Paulo – USP São Paulo SP Brazil; ^5^ Department of Health Sciences and Human Performance The University of Tampa Tampa Florida USA

**Keywords:** immunoblotting, myosin heavy chain, proteolysis, resistance exercise, skeletal muscle

## Abstract

**Abstract:**

We examined how resistance exercise (RE), cycling exercise and disuse atrophy affect myosin heavy chain (MyHC) protein fragmentation. The 1boutRE study involved younger men (*n* = 8; 5 ± 2 years of RE experience) performing a lower body RE bout with vastus lateralis (VL) biopsies being obtained prior to and acutely following exercise. With the 10weekRT study, VL biopsies were obtained in 36 younger adults before and 24 h after their first/naïve RE bout. Participants also engaged in 10 weeks of resistance training and donated VL biopsies before and 24 h after their last RE bout. VL biopsies were also examined in an acute cycling study (*n* = 7) and a study involving 2 weeks of leg immobilization (*n* = 20). In the 1boutRE study, fragmentation of all MyHC isoforms (MyHC_Total_) increased 3 h post‐RE (∼200%, *P* = 0.018) and returned to pre‐exercise levels by 6 h post‐RE. Interestingly, a greater magnitude increase in MyHC type IIa versus I isoform fragmentation occurred 3 h post‐RE (8.6 ± 6.3‐fold vs. 2.1 ± 0.7‐fold, *P* = 0.018). In 10weekRT participants, the first/naïve and last RE bouts increased MyHC_Total_ fragmentation 24 h post‐RE (+65% and +36%, *P *< 0.001); however, the last RE bout response was attenuated compared to the first bout (*P* = 0.045). Although cycling exercise did not alter MyHC_Total_ fragmentation, ∼8% VL atrophy with 2 weeks of leg immobilization increased MyHC_Total_ fragmentation (∼108%, *P *< 0.001). Mechanistic C_2_C_12_ myotube experiments indicated that MyHC_Total_ fragmentation is likely due to calpain proteases. In summary, RE and disuse atrophy increase MyHC protein fragmentation. Research into how ageing and disease‐associated muscle atrophy affect these outcomes is needed.

**Highlights:**

- **What is the central question of this study?**
  How different exercise stressors and disuse affect skeletal muscle myosin heavy chain fragmentation.
- **What is the main finding and its importance?**
  This investigation is the first to demonstrate that resistance exercise and disuse atrophy lead to skeletal muscle myosin heavy chain protein fragmentation in humans. Mechanistic in vitro experiments provide additional evidence that MyHC fragmentation occurs through calpain proteases.

## 1 INTRODUCTION

Our laboratory recently recruited college‐aged men with prior resistance training experience to perform two lower body resistance exercise (RE) bouts separated by 1 week consisting of 30% versus 80% one repetition loads (Sexton et al., 2023), these being termed 30‐FAIL and 80‐FAIL bouts, respectively. Vastus lateralis (VL) muscle biopsies were obtained immediately prior to as well as 3 and 6 h following these bouts, and we sought to holistically examine skeletal muscle molecular outcomes that differed between the two loading paradigms. Our first series of experiments indicated that both bouts similarly altered global DNA methylation and transcriptome‐wide markers (Sexton et al., 2023). Our second report indicated that both bouts similarly increased certain aspects of the mechanistic target of rapamycin signalling complex 1 (mTORC1) cascade while also similarly increasing follistatin mRNA and protein expression (McIntosh et al., 2023). Notably, both studies support prior literature suggesting that low‐ and high‐load training elicit similar post‐exercise anabolic signalling outcomes so long as sets are performed near failure (Haun et al., 2017; Mitchell et al., 2012; Morton et al., 2019).

The final phase of project analysis began with utilizing the remaining 30‐FAIL tissue for eight participants to examine if titin phosphorylation was altered 3 or 6 h following exercise. Our interest was spawned by past reviews suggesting this phenomenon may be a catalyst for post‐exercise anabolic signalling (Kruger & Kotter, 2016; Wackerhage et al., 2019). To accomplish this aim, myofibrils were isolated and solubilized using our recently published myofibril isolation and solubilisation technique (MIST) method adopted by us and others (Baehr et al., 2021; Olsen et al., 2023; Oxfeldt et al., 2023; Roberts et al., 2020). During pilot SDS‐PAGE Coomassie experiments with myofibril isolates, we observed notable protein fragmentation occurred 3 h following exercise that was visibly reversed by the 6 h post‐exercise time point (depicted in Figure 2 in the Results section). With preliminary immunoblotting experiments we also observed a similar trend with the titin protein, thus precluding phosphorylation analysis. After a thorough examination of the literature, only one paper has reported that a RE bout promotes titin and myosin heavy chain (MyHC) fragmentation 3 h following a RE bout (Wette et al., 2021), and this report was in seven previously trained men. However, aside from briefly mentioning this as being potential evidence of protein disruption with resistance training, the significance of this finding was not further explored. It is well known that skeletal muscle tissue is ∼90% spatially occupied by myofibres (Roberts et al., 2023), and that each myofibre is volumetrically occupied by ∼85% myofibrils. Accordingly, MyHC and actin are the most abundant proteins in skeletal muscle, and a proteomic analysis by our laboratory supports this, indicating that MyHC is the most abundant protein (∼42%) in the muscle protein pool (Vann, Roberson et al., 2020). Given MyHC's importance and abundance, its fragmentation is notable as it may provide insight into the remodelling process.

Our preliminary observations motivated a series of exploratory experiments using VL biopsy specimens from various human studies. Data from one study (termed ‘1boutRE’) provides compelling evidence that significant MyHC fragmentation occurs 3 h following a single lower body RE bout in well‐trained males. However, MyHC fragments are largely absent 6 h following exercise, implying that skeletal muscle can rapidly clear these proteins following a loading stimulus. In a second study (termed ‘10weekRT’), we observed that MyHC fragmentation is present 24 h following a leg extensor bout in a large cohort of untrained males and females, and that this 24 h response is attenuated after 10 weeks (24 total sessions) of leg extensor resistance training. Results from our third study indicated that 60 min of cycling exercise did not promote MyHC fragmentation 2 or 8 h post‐exercise. Results from our fourth study indicated that 2 weeks of disuse atrophy through leg immobilization promoted a robust MyHC fragmentation response. We believe that this easy‐to‐perform immunoblot‐based technique could be used as a proxy marker of protein degradation in RE studies or disuse studies. Experimental details and an expanded discussion of these findings are provided in the following paragraphs.

## 2 METHODS

### 2.1 Ethical approval for human studies

Verbal and written informed consent were obtained for all human studies. Institutional Review Board (IRB) protocol numbers as well as clinical database registry identifiers (when applicable) are provided in each of the paragraphs below describing study participants. Two studies (1boutRE, cycling study) conformed to the standards set by the *Declaration of Helsinki*, except for registration in a database. Two studies (10weekRT, Leg immobilization) conformed to the standards set by the *Declaration of Helsinki* and were pre‐registered as clinical trials.

### 2.2 1boutRE study participants

**FIGURE 1:**
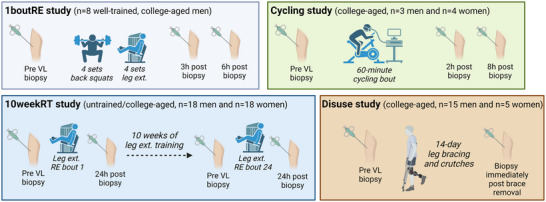
Summary of human studies. Schematic (drawn using Biorender.com) illustration of the study logistics and participant number for each human study whereby MyHC analyses occurred. More details related to each study can be found in‐text.

Muscle specimens from well‐trained college‐aged males (*n* = 8; 22 ± 3 years old, 5 ± 2 years of RE experience, 83.0 ± 7.0 kg, 1.6 ± 0.3 one repetition maximum squat: body mass) as described by Sexton et al. (2023), and all experimental procedures were approved by the Auburn University Institutional Review Board (IRB protocol no. 20‐081). Information regarding participant characteristics, the acute lower body RE exercise bout, and the procurement of VL biopsies can be found in Sexton et al. (2023). Briefly, participants reported to the laboratory during morning hours in a fasted state. After donating a baseline biopsy, participants performed four sets each of the back squat and leg extension exercises at 30% of their estimated one‐repetition maximum loads until volitional failure. Five minutes of rest was allowed between sets and exercises. Following the RE bout, VL biopsies were collected 3 and 6 h post‐exercise. This, along with other human studies detailed in the following paragraphs are visually depicted in Figure 1.

### 2.3 10weekRT study participants

Muscle specimens were from healthy college‐aged participants (*n* = 38 total with 19 women (24.2 ± 4.9 years old, 62.7 ± 8.5 kg, and 1.64 ± 0.1 m) and 19 men (24.5 ± 3.3 years old, 73.6 ± 13.4 kg, 1.76 ± 0.1 m)) as previously described by Scarpelli et al. (2024b). Due to sample limitations for two participants, only 36 participants were analysed. All experimental procedures were approved by the local ethics committee, the study was conducted in accordance with the most recent version of the *Declaration of Helsinki* and was registered as a clinical trial (Brazilian Registry of Clinical Trials – RBR‐57v9mrb), and training as well as specimen collection was performed at the University of Sao Carlos. The resistance training protocol consisted of four sets of 9−12 maximum repetitions of unilateral leg extension exercises, with a 90 s rest period between sets. The load was adjusted for each set to ensure that concentric muscle failure occurred within the target repetition range. Participants completed 24 training sessions over a period of 10 weeks, with sessions conducted two to three times per week. Critically, four mid‐thigh VL biopsies were obtained before (Pre) and 24 h after the first training bout (untrained state), 96 h after the second to last training bout (Pre), and 24 h after the last training bout (trained state).

### 2.4 Cycling study and leg immobilization study participants

To determine how an acute cycling bout affects post‐exercise MyHC fragmentation, human muscle specimens from a previously published study from our laboratory were analysed (IRB protocol no. 18‐226) (Roberson et al., 2019). To determine how non‐complicated (i.e., without injury or illness) disuse atrophy affects MyHC fragmentation, human muscle specimens from another ongoing Auburn University IRB‐approved study (IRB protocol no. 23‐220, clinicaltrials.gov study identifier NCT05760066) were analysed. Experimental procedures from both studies were approved by the Auburn University Institutional Review Board and were conducted at Auburn University.

For the cycling study, apparently healthy college‐aged participants (*n* = 7, three males and four females; 23 ± 3 years old, 23.0 ± 2.9 kg/m^2^) reported to the laboratory during the morning hours under fasted conditions and donated a baseline VL biopsy. Participants then mounted a cycle ergometer (Velotron, RacerMate, Seattle, WA, USA) and performed a 5‐min warm‐up at a self‐selected pace. Wattage was adjusted thereafter to achieve 70% ${\dot{V}}_{O_{2}}$ reserve and participants cycled for 60 min. Post‐exercise biopsies were then obtained 2 and 8 h following the cycling bout.

For the leg immobilization study, apparently healthy college‐aged participants (*n* = 20, 15 males and five females; 26 ± 3 years old, 25.9 ± 5.6 kg/m^2^) reported to the laboratory under fasted conditions and donated a baseline VL biopsy from their left leg. Participants’ left legs were then fitted with a knee brace locked at 90° and administered crutches and explicit instructions to prevent weight‐bearing activities for a 14‐day period. Following the 14‐day disuse period, participants reported back to the laboratory under fasted conditions (±2 h from the first visit) and donated a second VL biopsy from the left leg ∼1–2 cm from the previous biopsy incision. Left leg mid‐thigh VL muscle cross sectional area (mCSA) measurements were also obtained at each visit via panoramic ultrasound imaging using methods previously described by multiple studies by Ruple and colleagues (Ruple, Mesquita et al., 2022, Ruple, Smith et al., 2022b). Briefly, mid‐thigh was determined by measuring the total distance from the mid‐inguinal crease in a straight line to the proximal patella, with the hip and knee flexed at 90°. A mark at the location corresponding to 50% of the total length was then made using a marker. This mark was used to capture all pre‐intervention ultrasound images and as well as the pre‐intervention biopsy. A multifrequency linear‐array transducer (L4‐12T, 4−12 MHz; GE Healthcare, Chicago, IL, USA) was used to capture VL images in the transverse plane (cross‐sectional orientation), and a flexible/semirigid pad was placed around the thigh and secured with an adjustable strap. The pad was used solely as a guide to ensure probe movement was confined to the transverse plane, and scans began at the lateral aspect of the thigh moving medially until the rectus femoris was visualized. Images were captured using the panoramic function of the device (LogicView, GE Healthcare) and all ultrasound settings were held constant across participants and laboratory visits (frequency: 10 MHz, gain: 50 dB, dynamic range: 75). Images were downloaded and analysed offline using the freely available ImageJ software (National Institutes of Health, Bethesda, MD, USA). Importantly, the pre‐intervention biopsy scar was used as the location for post‐immobilization ultrasound image collection.

### 2.5 1boutRE tissue myofibril and cytoplasmic fractionation

Using a liquid nitrogen‐cooled ceramic stage, approximately 20 mg of muscle from each biopsy specimen was placed in 1.7 mL tubes containing 300 µL of ice‐cold homogenizing buffer (Buffer 1: 20 mM Tris–HCl, pH 7.2, 5 mM EGTA, 100 mM KCl, 1% Triton X‐100; all chemicals from VWR; Radnor, PA, USA). Samples were homogenized using tight‐fitting pestles and centrifuged at 3000 *g* for 30 min at 4°C. Supernatants (cytoplasmic fraction) were transferred to new 1.7 mL tubes and stored at −80°C until protein analyses described below. As a wash step, resultant pellets (myofibrillar fraction) were resuspended in Buffer 1, and samples were centrifuged at 3000 *g* for 10 min at 4°C. Resultant supernatants from this step were discarded, myofibril pellets were resuspended in 300 µL of ice‐cold wash buffer (Buffer 2: 20 mM Tris–HCl, pH 7.2, 100 mM KCl, 1 mM dithiothreitol (DTT); all chemicals from VWR), and tubes were centrifuged at 3000 *g* for 10 min at 4°C; this step was performed twice. Final myofibril pellets were resuspended in 400 µL of ice‐cold storage buffer (Buffer 3: 20 mM Tris–HCl, pH 7.2, 100 mM KCl, 20% glycerol, 1 mM DTT, 50 mM spermidine; all chemicals from VWR), and stored at −80°C for analyses described below.

### 2.6 Whole tissue lysate preparations for 10weekRT, cycling, and leg immobilization studies

Using a liquid nitrogen‐cooled ceramic stage, approximately 20 mg of muscle from each biopsy specimen was placed in 1.7 mL tubes containing 400 µL of commercially available general cell lysis buffer (Cell Signaling Technology; Danvers, MA, USA; cat. no.: 9803). The lysis buffer contained 20 mM Tris–HCl (pH 7.5), 150 mM NaCl, 1 mM Na_2_EDTA, 1 mM EGTA, 1% Triton X‐100, 2.5 mM sodium pyrophosphate, 1 mM β‐glycerophosphate, 1 mM Na_3_VO_4_, and 1 µg/mL leupeptin. Samples were centrifuged at 500 *g* for 5 min at 4°C. Resultant supernatants from placed in new 1.7 mL tubes and stored at −80°C for analyses described below.

### 2.7 MyHC immunoblotting

Protein concentrations of 1boutRE myofibril and cytoplasmic isolates, and whole tissue lysates from the other studies were quantified using bicinchoninic acid (BCA) colorimetric assay kits (Thermo Fisher Scientific, Waltham, MA, USA). Isolates and lysates from all studies were then prepared for western blot analysis with 4× Laemmli buffer for final concentration preparations at 1 µg/µL. Aliquots of prepared samples (4 µL for myofibrillar preps, 15 µL for cytoplasmic preps, and 10 µL of whole tissue lysate preps) were applied to 4–15% SDS‐polyacrylamide gels (Bio‐Rad Laboratories; Hercules, CA, USA) and subjected to electrophoresis at 180 V for 50 min in a preformulated 1× SDS‐PAGE buffer (VWR). Proteins were then electrotransferred onto pre‐activated polyvinylidene difluoride membranes (Bio‐Rad) for 2 h on ice, Ponceau stained, and placed in a gel documentation system (ChemiDoc Touch; Bio‐Rad) to capture whole‐lane images for protein normalization purposes. Membranes were then blocked in a solution containing 5% skimmed milk powder in Tris‐buffered saline with 0.1% Tween‐20 (VWR) for 1 h at ambient temperature.

Incubation of membranes with anti‐MyHC antibodies was carried out for 1–2 h (room temperature) at a dilution of 1:200 in Tris‐buffered saline with 0.1% Tween‐20 (TBST) containing 5% bovine serum albumin. These antibodies included non‐concentrated clone supernatants of: (i) mouse monoclonal IgG2a MyHC, termed MyHC_Total_ throughout (Developmental Studies Hybridoma Bank; Iowa City, IA, USA; cat. no.: A4.1025), (ii) mouse monoclonal IgG1 MyHCI (Developmental Studies Hybridoma Bank; cat. no.: A4.951), (iii) mouse monoclonal IgG1 MyHCIIa (Developmental Studies Hybridoma Bank; cat. no.: SC‐71), and (iv) mouse monoclonal IgM MyHCIIx (Developmental Studies Hybridoma Bank; cat. no.: 6H1). Following primary antibody incubations, membranes were washed for 15 min in TBST and incubated with horseradish peroxidase‐conjugated anti‐mouse IgG (Cell Signaling Technology; cat. no.: 7076) or IgM (Thermo Fisher Scientific; cat. no.: 31440) secondary antibodies at a dilution of 1:2000 in TBST with 5% BSA for 1 h (room temperature) prior to development steps described below.

Membranes were developed for 1–5 s using an enhanced chemiluminescence reagent (Luminata Forte HRP substrate; Millipore Sigma, Burlington, MA, USA) in a gel documentation system (ChemiDoc Touch; Bio‐Rad). The densitometry of prominent MyHC bands and fragments were quantified with ImageLab v6.0.1 (Bio‐Rad) using the ‘Lanes & Bands Tool’ functions. Densitometry readings for bands and targets were normalized to baseline (Pre) values, which were averaged to a value of 1.00, and expressed as fold‐change from Pre.

### 2.8 Immunoprecipitation for MyHC_Total_ polyubiquitination in 1boutRE myofibril isolates

To determine if MyHC fragments were polyubiquitinated, immunoprecipitation (IP) experiments were performed on 1boutRE myofibril isolates (resuspended in Buffer 3 described in Section 2.5) using a commercially available kit (Dynabeads Protein G; Thermo Fisher Scientific; cat. no.: 10009D). Per sample reaction, 50 µL of resuspended bead slurry was mixed with 30 µL of mouse monoclonal IgG2a anti‐MyHC (Developmental Studies Hybridoma Bank; cat. no.: supernatant of A4.1025) for 60 min at room temperature on an inversion apparatus. Bead‐IgG2a complexes were washed with antibody binding/wash buffer provided by the kit and subsequently incubated with 600 µg of myofibril protein per sample for 60 min at room temperature on an inversion apparatus. Bead‐Ab‐Ag complexes were then washed three times with wash buffer provided by the kit, and 20 µL of elution buffer as well as 10 µL of 4× Laemmli buffer was added. Samples were boiled for 5 min at 100°C, beads were removed using a magnetic rack apparatus, and immunoblotting experiments were carried out on 10 µL of resultant IP preps whereby polyubiquitinated MyHC fragments were probed using a polyclonal rabbit IgG antibody (1:1000; Cell Signaling Technology; cat. no.: 3933). In addition to these IP experiments, 1boutRE myofibril isolates were immunoblotted for polyubiquitination using the same polyclonal rabbit IgG antibody and immunoblotting methods described in the prior section.

### 2.9 Cell culture

Mechanistic in vitro experiments were performed to better determine if MyHC fragmentation is due to calpain activity. C_2_C_12_ immortalized murine myoblasts (ATCC, Manassas, VA, USA), passage number 8, were seeded at ∼3 × 10^5^ cells per well in six‐well plates containing 3 mL/well of growth medium (GM) consisting of Dulbecco's modified Eagle's medium (DMEM; Corning, Corning, NY, USA), 10% fetal bovine serum (FBS; Corning), 1% penicillin/streptomycin (VWR), 0.1% gentamycin (VWR). Upon reaching confluency (∼80–90%), myoblasts were differentiated into myotubes by switching to differentiation medium (DM) containing DMEM, 2% horse serum (VWR), 1% penicillin/streptomycin (VWR), and 0.1% gentamycin (VWR). DM was replaced every 48 h until confluency of mature myotubes was achieved (∼7 days). Myotubes (six replicate wells) were treated for 24 h with one of serum‐free DMEM (atrophy stimulus), serum‐free DMEM + 50 µM calpeptin (Enzo Life Sciences, Farmingdale, NY, USA; cat. no.: 89161–562), DM+20% FBS (anabolic stimulus), or DM+20% FBS + 50 µM calpeptin. Our laboratory has reported that the utilized calpeptin dose (6 h treatment) significantly reduces C_2_C_12_ myotube calpain activity by ∼80% (Osburn et al., 2021).

Aside from brief media exchanges, plates were maintained in incubators set to 37°C and 5% CO_2_ at all phases of experimentation. Following 24 h treatments (15 min prior to collection), cells were pulse labelled with 1 mM puromycin dihydrochloride (VWR; cat. no.: 97064–280) to assess relative muscle protein synthesis (MPS) responses using the SUnSET method (Goodman et al., 2011). Cells were then collected using ice‐cold general cell lysis buffer (Cell Signaling Technology; cat. no.: 9803). Lysates were processed for total protein using the BCA protein assay kit (Thermo Fisher Scientific) and spectrophotometer (Agilent Biotek Synergy H1 hybrid reader; Agilent Technologies, Santa Clara, CA, USA). Calpain activity assays were performed using chemiluminescence assays (Promega Corp., Madison, WI, USA; cat. no.: G8502) similar to methods previously published by our laboratory (Scarpelli et al., 2024a), MyHC_Total_ fragmentation was assessed using western blotting methods described in prior sections, and relative muscle protein synthesis levels were assessed using western blotting methods with a mouse anti‐puromycin antibody (1:10,000, Millipore Sigma; cat. no.: MABE342).

Myotubes from select treatment groups were also immuno‐stained to assess morphology. Briefly, myotubes were fixed with 10% formalin (VWR) for 15 min at room temperature then washed 3 × 3 min with phosphate‐buffered saline (PBS) containing 0.2% Triton X‐100 (PBS/Triton). Cells were then blocked with PBS/Triton containing 1% BSA for 1 h at room temperature followed by incubation with a primary antibody solution containing anti‐MyHC (1:100) (Developmental Studies Hybridoma Bank; cat. no.: A4.1025) in PBS/Triton/BSA for 3 h at room temperature. Following 3 × 3‐min washes with PBS/Triton, cells were incubated with a secondary antibody solution containing goat anti‐mouse IgG2a AF488 (Thermo Fisher Scientific; cat. no.: A‐21131) in PBS/0.2% Triton X‐100 for 2 h at room temperature. Cells were then washed 3 × 3 min with PBS/Triton and incubated with 4′,6‐diamidino‐2‐phenylindole (DAPI) (Thermo Fisher Scientific; cat. no.: D3571) for 10 min. After the last wash, multiple images were obtained by a fluorescence microscope using a ×10 objective (Zeiss Axio imager M2; Zeiss Microscopy, Jena, Germany).

This treatment schematic was based on the hypothesis that serum starvation (serum‐free DMEM) would promote myotube atrophy (Goodman et al., 2011), as well as enhanced MyHC_Total_ fragmentation via protein breakdown. Additionally, we hypothesized that serum stimulation (DM+20% FBS) would lead to increased protein synthesis and myotube hypertrophy (Stec et al., 2016), as well as diminished calpain‐mediated proteolysis and MyHC_Total_ fragmentation. Finally, we sought to determine if calpain activity inhibition with 50 µM calpeptin, as previously demonstrated by our laboratory (Osburn et al., 2021), reduced MyHC_Total_ fragmentation in the event that this process was stimulated by either serum starvation or serum stimulation.

### 2.10 Statistical analysis

Statistics were performed and graphs were constructed using commercially available software (GraphPad Prism, v10.1.0; GraphPad Software, Boston, MA, USA). Most 1boutRE and all cycling study data were analysed via one‐way repeated measures ANOVA. When significant model effects were observed (*P *< 0.05), Tukey's post‐hoc test was performed to determine which time points were significantly different from one another. The only 1boutRE data analysed via two‐way repeated measures ANOVAs were isoform‐specific data. When significant model effects were observed (*P *< 0.05), Fisher's LSD post‐hoc test was performed to determine which time points were significantly different from one another. All 10weekRT data were analysed via two‐way (training status × time) repeated measures ANOVA. When significant model effects were observed (*P *< 0.05), Tukey's post‐hoc test was performed to determine which time points were significantly different from one another. Leg immobilization study data were analysed using Student's *t*‐test for dependent samples. Finally, in vitro data were analysed using two‐way ANOVA (±serum, ±calpeptin) to unveil main treatment effect *P*‐values. Data throughout are presented as means and standard deviation with individual data points.

## 3 RESULTS

### 3.1 Evidence of post‐exercise myofibril protein fragmentation following a single RE bout

**FIGURE 2:**
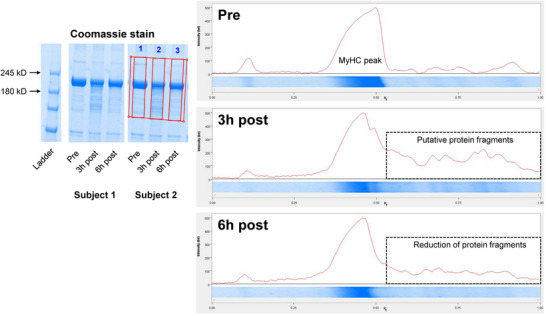
Evidence of post‐exercise myofibril protein fragmentation in 1boutRE study participants. As discussed in‐text, preliminary 1boutRE experiments were performed on two well‐trained participants’ myofibril isolates aiming to examine the presence of titin using 4–15% SDS‐PAGE gels and Coomassie staining. In both participants, visual myofibril protein fragments were observed in the myosin heavy chain (MyHC) kilodalton region 3 h following the resistance exercise bout. Conversely, the rapid disappearance of these fragments was evident by the 6 h post‐exercise time point.

As noted in the Introduction section, our experiments began with attempting to interrogate the titin protein from 1boutRE specimens. Figure 2 shows preliminary SDS‐PAGE Coomassie experiments on myofibril isolates in two participants from this study. Notably, visual protein fragments were observed in the MyHC region 3 h following the RE bout, and the rapid disappearance of these fragments was evident by 6 h post‐exercise.

### 3.2 Transient post‐exercise MyHC_Total_ fragmentation following a single RE bout

**FIGURE 3:**
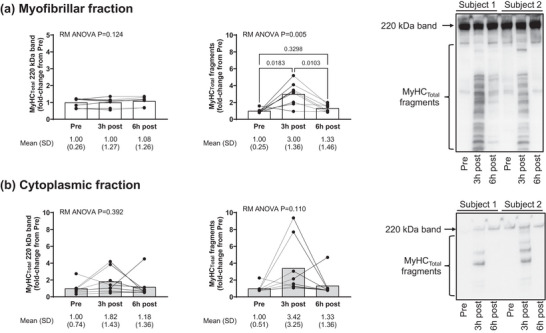
Post‐exercise MyHC_Total_ fragmentation in 1boutRE participants. (a) Data from well‐trained 1boutRE men (*n* = 8) show that significant total myosin heavy chain (MyHC_Total_) fragmentation is evident in the myofibril fraction 3 h following a resistance exercise bout; however, the rapid (and significant) disappearance of these fragments was evident by the 6 h post‐exercise time point. (b) Also notable is the high presence of MyHC_Total_ fragments in the cytoplasmic fraction in several participants; however, this did not reach statistical significance. Representative immunoblots are shown for two of eight participants, and data are presented as mean and SD values with repeated measures (RM) ANOVA *P*‐values.

Figure 3 shows MyHC_Total_ immunoblotting experiments in all 1boutRE participants. The increased presence of MyHC_Total_ fragmentation was evident in the myofibril fraction 3 h following RE, and the rapid disappearance of these fragments was evident by the 6 h post‐exercise time point (Figure 3a). MyHC_Total_ fragmentation was also observed at the 3 h post‐exercise time point in the cytoplasmic fraction of several participants (Figure 3b), albeit this did not reach statistical significance.

### 3.3 Isoform‐specific MyHC fragmentation following a single RE bout

**FIGURE 4:**
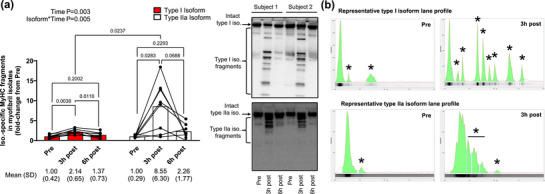
Type I versus IIa MyHC isoform fragmentation in 1boutRE participants. (a) Data from well‐trained 1boutRE men (*n* = 8) show that significant MyHC fragmentation of the type I and IIa isoforms is evident in the myofibril fraction 3 h following the resistance exercise bout; however, as with MyHC_Total_ fragments, the rapid (and significant) disappearance of I and IIa fragments was evident by the 6 h post‐exercise time point. Also notable were the different patterns of fragmentation between isoforms, with lower molecular mass type I isoform fragments appearing post‐RE versus heavier type IIa fragments. Representative immunoblots are shown for two of eight participants, and data are presented as means and SD values with two‐way (isoform × time) ANOVA time and interaction *P*‐values. (b) Lane profiles of type I and IIa isoform fragmentation from two different participants where ‘*’ indicates fragments detected by analysis software.

Figure 4 shows isoform specific MyHC immunoblotting experiments in all 1boutRE participants. The increased presence of type I and IIa MyHC fragments was evident in the myofibril fraction 3 h following the RE bout, and the rapid disappearance of these fragments was evident by the 6 h post‐exercise time point (Figure 4a). Additionally, the magnitude of 3 h post‐RE type IIa isoform fragmentation was greater than type I isoform fragmentation (*P* = 0.024). Finally, there were visually different patterns of fragmentation between isoforms, with lower molecular mass type I isoform fragments appearing post‐RE versus heavier type IIa fragments (Figure 4b).

### 3.4 Polyubiquitination of myofibril proteins and MyHC_Total_ poly‐ubiquitination following a single RE bout

**FIGURE 5:**
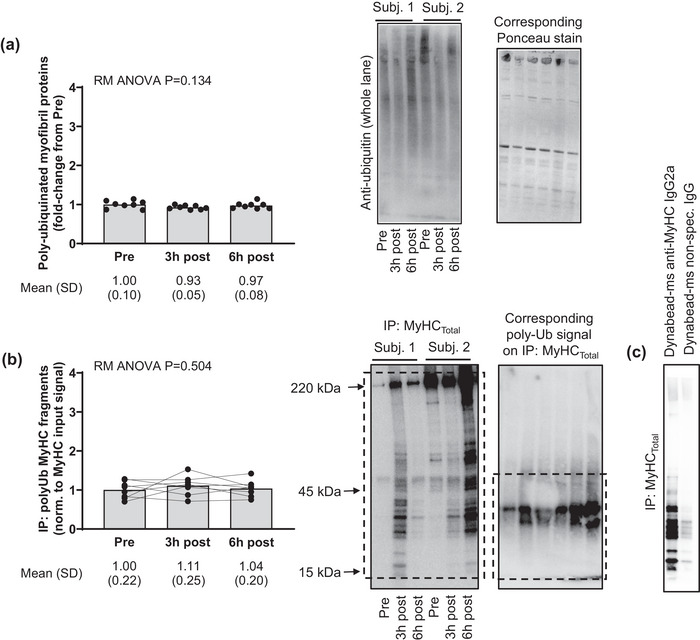
Total myofibril protein and MyHC_Total_ poly‐ubiquitination in 1boutRE participants. (a) Data from well‐trained 1boutRE men (*n* = 8) show that total myofibril protein polyubiquitination levels remain unaltered post‐exercise. (b) Additionally, the polyubiquitination signal on immunoprecipitated MyHC fragments (spanning ∼15–50 kDa) remained unaltered 3 and 6 h post‐exercise when data were normalized to the IP: MyHC signal. (c) The Dynabead‐mouse anti‐MyHC IgG2a yielded an appreciably higher signal of putative MyHC fragments compared to a negative control (Dynabead‐mouse non‐specific IgG) when incubated with ∼600 µg myofibril isolates from the same participant sample. Representative immunoblots are shown for two of eight participants, and data are presented as means and SD values with one‐way repeated measures (RM) ANOVA *P*‐values. MyHC, myosin heavy chain.

Figure 5 shows myofibril protein polyubiquitination and MyHC_Total_ polyubiquitination in all 1boutRE participants. Myofibril protein poly‐ubiquitination levels did not significantly differ between pre‐ and post‐exercise time points (Figure 5a). Moreover, polyubiquitinated fragments in the ∼15–50 kDa region (where the signal was prominent) were not significantly altered when this signal was normalized to the MyHC IP signal (Figure 5b). Also notable is the lack of polyubiquitinated MyH_Total_ fragments in the ∼50–220 kDa region.

### 3.5 Post‐exercise MyHC_Total_ fragmentation in 10weekRT participants in the naïve and trained states

**FIGURE 6:**
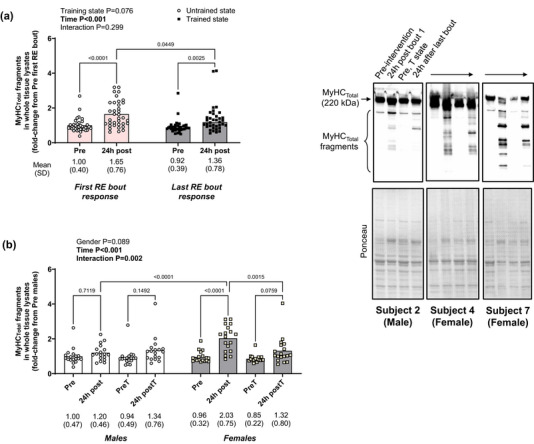
Twenty four hour post‐exercise MyHC_Total_ fragmentation in the untrained and trained states in 10weekRT participants. (a) Data from all 10weekRT participants (*n* = 36) show that significant total myosin heavy chain (MyHC_Total_) fragmentation is evident in the whole tissue lysate 24 h following the first/naïve resistance exercise bout. While this same 24 h post‐exercise response occurs following 10 weeks of training (24 leg extensor sessions), it is significantly attenuated. (b) Sex analysis in 10weekRT participants (18 men and 18 women) show that the 24 h first bout RE responses in (a) are largely driven by females. Representative immunoblots are shown for three participants, and data are presented as means and SD values with two‐way (training state × time) ANOVA main effect and interaction *P*‐values.

Figure 6 shows MyHC_Total_ fragmentation responses in 10weekRT participants. Significant increases were observed 24 h following the first/naïve and last training bouts (Figure 6a). However, this response was attenuated following the last versus the first/naïve bout (*P* = 0.045).

Given that there was a robust number of men and women in this study (*n* = 18 per sex), we also examined MyHC_Total_ fragmentation responses between sexes (Figure 6b). Interestingly, a two‐way (sex × time) repeated measures ANOVA indicated that significant 24 h MyHC_Total_ fragmentation following the first/naïve bout was only evident in females (*P *< 0.001 within and between sexes). However, this response in females was attenuated 24 h following the last bout of training (*P* = 0.002).

### 3.6 MyHC_Total_ fragmentation is absent following a cycling bout

**FIGURE 7:**
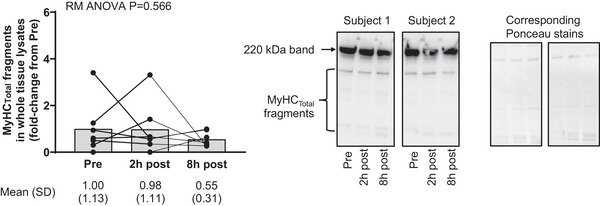
MyHC_Total_ fragmentation is absent following a cycling exercise bout. Data from cycling study participants (*n* = 7) show that MyHC_Total_ fragmentation is not significantly altered 2 and 8 h following a 60‐min cycling exercise bout. Representative immunoblots are shown for two participants. Data are presented as means and SD values with repeated measures (RM) one‐way ANOVA *P*‐value. MyHC_Total_, total myosin heavy chain.

Figure 7 shows MyHC_Total_ fragmentation responses in the seven participants who engaged in 60 min of cycling exercise. Unlike what was observed with the RE responses, there was a lack of post‐exercise fragmentation 2 and 8 h following the cycling bout.

### 3.7 MyHC_Total_ fragmentation increases following 2 weeks of leg immobilization

**FIGURE 8:**
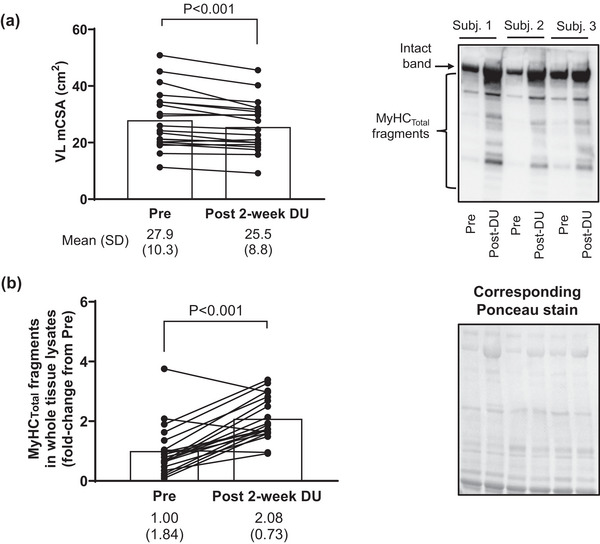
MyHC_Total_ fragmentation increases following 2 weeks of disuse atrophy. (a) Data from 2‐week disuse participants (*n* = 20) show that VL muscle atrophy occurs with lower‐limb immobilization (determined by ultrasound). (b) This coincides with significant MyHC_Total_ fragmentation. Representative immunoblots are shown for three participants, and data are presented as means and SD values with dependent *t*‐test *P*‐values. MyHC_Total_, total myosin heavy chain.

Figure 8 shows data in the 20 participants who underwent leg immobilization for 2 weeks. Leg immobilization led to ∼8% mid‐thigh VL atrophy (*P *< 0.001, Figure 8a) and this coincided with a 108% increase in MyHC_Total_ fragmentation (*P *< 0.001, Figure 8b).

Although there were appreciably more men than women in this cohort (15 vs. 5, respectively), statistical analyses were still performed to determine if responses were similar between sexes. Following disuse, MyHC_Total_ fragmentation increased in men (92%, *P* = 0.002) and women (164%, *P *< 0.001), and while the magnitude was greater in women, these responses were statistically similar between sexes (*P* = 0.374). Likewise, no difference in VL muscle atrophy was apparent between sexes (men: −8.7 ± 7.5%, women: −5.4 ± 3.4%, *P* = 0.355).

### 3.8 Evidence in myotubes suggests calpains promote MyHC_Total_ fragmentation

**FIGURE 9:**
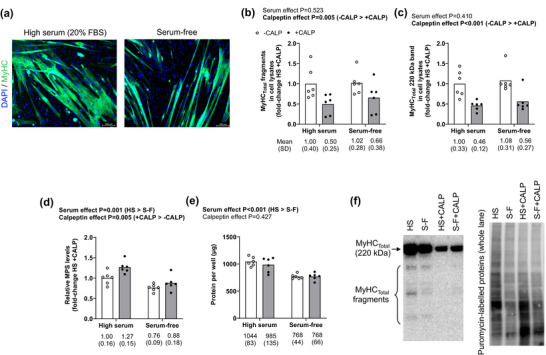
C_2_C_12_ myotube data demonstrating MyHC_Total_ fragmentation is due to calpains. (a) Representative microscopy images of C_2_C_12_ myotubes treated with or without 20% fetal bovine serum as well as with or without calpeptin (50 µM, calpain inhibitor) for 24 h (×10, scale bar 100 µm). (b, c) Calpeptin treatments reduced MyHC_Total_ fragmentation (b) as well as the presence of intact MyHC protein (c). (d, e) Calpeptin treatments also paradoxically increase protein synthesis levels (d) without affecting protein yield (e). Tangential findings include high serum treatments being more anabolic than serum‐free treatments as evidenced through heightened protein synthesis levels (d) and protein yields (e). (f) Representative immunoblots of data presented in figures. Data are presented as means and (SD) values with treatment effect *P*‐values. MyHC_Total_, total myosin heavy chain.

Figure 9 shows in vitro C_2_C_12_ myotube data from different 24 h treatments. Serum‐free treated myotubes (regardless of calpain inhibition) demonstrated an atrophic phenotype as evidenced by smaller myotube diameters (Figure 9a), reduced protein synthesis levels (*P* = 0.001, Figure 9d), and lower protein yields per well (*P *< 0.001, Figure 9e). Moreover, calpeptin treatments (regardless of serum condition) reduced the presence of MyHC_Total_ fragmentation (*P* = 0.005, Figure 9b) implicating the role of calpains in this process. However, there were findings paradoxical to our hypotheses. First, serum‐free medium, while causing atrophy, did not promote MyHC_Total_ fragmentation. Moreover, calpeptin treatments reducing intact MyHC protein content (*P *< 0.001, Figure 9c) while increasing protein synthesis levels (*P* = 0.005, Figure 9d).

## 4 DISCUSSION

This investigation provides evidence that RE and disuse in humans promote MyHC fragmentation. Additionally, while there are limitations to the employed in vitro model, our data suggest MyHC fragmentation operates through calpain‐mediated proteolysis. The absence of MyHC fragmentation following a cycling bout implies this process is likely at least partially mediated by mechanical loading. These findings are physiologically relevant given that the MyHC protein is needed for proper muscle function and is by far the most abundant protein in skeletal muscle (i.e., ∼42% of the total muscle protein pool in college‐aged men according to our recent proteomic estimates; Vann, Roberson et al., 2020). As mentioned prior, only one other study has reported that a RE bout increases titin and MyHC fragmentation in whole tissue lysates 3 h following exercise, in seven previously trained men (Wette et al., 2021). Although the authors did not extensively pursue the significance of this finding, other studies indirectly support that the fragmentation of MyHC and other myofibrillar proteins likely occur following a RE bout. For example, Beaton et al. (2002) demonstrated a loss of sarcomeric structural proteins such as desmin 4 and 24 h following a bout of eccentric exercise in recreationally trained men. Nielsen et al. (2015) reported that a bout of eccentric loading leads to significant z‐/m‐line disruption 3, 24 and 48 h following exercise in untrained men; notably, z‐/m‐line disruption is an ultrastructural feature that may represent the release of myofilaments from intracellular structures (Friden & Lieber, 2001). Phillips et al. (1997) reported that muscle protein breakdown (MPB) rates following RE peak at 3 h in untrained men, and a significant elevation in MPB is also evident in these individuals 24 h following exercise. These researchers have also reported that MPB rates are significantly elevated in resistance trained men 4 h following a leg RE bout (Phillips et al., 1999).

Ample availability of 1boutRE biopsy specimens allowed for a more expanded analysis relative to the other studies. Aside from isoform specific fragmentation patterns (discussed in the next paragraph), myofibril isolates were also analysed for MyHC protein polyubiquitination to potentially explain the rapid disappearance of fragments by the 6 h post‐RE time point. The muscle RING finger 1 (MuRF1) and muscle atrophy F‐box protein (MAFbx) E3 ligases catalyse sarcomeric protein poly‐ubiquitination for degradation through the proteasome (Clarke et al., 2007; Lokireddy et al., 2011), although there is additional evidence that polyubiquitinated protein aggregates can undergo selective lysosome degradation (Paudel et al., 2023). Hence, we hypothesized that post‐RE MyHC fragments are likely polyubiquitinated. Contrary to this hypothesis, however, were the IP experiment results indicating that polyubiquitinated MyHC_Total_ fragments (normalized to the MyHC_Total_ IP signal) were not altered 3 and 6 h post RE. Moreover, polyubiquitinated myofibril proteins were not altered at select post‐exercise time points and there was a lack of polyubiquitinated MyHC_Total_ fragments in the ∼50–220 kDa range. One limitation to consider with this analysis was the lack of inhibitors (e.g., *N*‐ethylmaleimide) that protect protein ubiquitylation in buffers used to isolate myofibrils. Hence, there is a small possibility that MyHC–ubiquitin complexes could have been dissociated with tissue lysis during myofibril isolation. However, data in Figure 5a show that the myofibril protein pool presented a polyubiquitination signature. Moreover, previous data published by our laboratory demonstrate that the ubiquitin antibody used in the current study readily detects proteolytic activity as observed by the accumulation of polyubiquitinated proteins in myotubes treated with a proteasome inhibitor (Osburn et al., 2021). Hence, this lends further credence that post‐RE MyHC fragments are not polyubiquitinated and may be cleared from muscle through non‐proteolytic mechanisms. These findings call into question how MyHC fragments are cleared from muscle. It is tempting to speculate that MyHC fragments have dissociated peptide bonds rapidly repaired and are re‐associated back into myofibrils post‐RE. However, enzymes facilitating this process require a catalytic domain that possesses peptide bond formation capabilities, and although non‐ribosomal peptide synthetases exist in bacteria (Miller & Gulick, 2016), these enzymes are not present in mammalian cells. Another explanation is that MyHC fragments are packaged into extracellular vesicles (EVs) and are released into circulation post‐RE. This is not too far‐fetched given that others have reported robust elevations in circulating EVs immediately post‐RE (Conkright et al., 2022), and MyHC has been reported to be enriched in circulating EVs (Whitham et al., 2018). However, we were not able to test this hypothesis given that blood was not obtained in 1boutRE participants. Therefore, future research is needed in further determining the fate of post‐RE MyHC fragments.

The unique RE‐induced MyHC I and IIa isoform fragmentation responses in the 1boutRE study participants also warrant consideration. Although the isoform responses were indeed interesting, this finding was not unanticipated given that fibre type‐specific macromolecule and proteome differences have been reported (reviewed in Roberts et al., 2023). Calpains, which are chiefly responsible for the cleavage of MyHC (Murphy, 2010), have been reported to be differentially expressed in slow‐ versus fast‐twitch muscle (Jones et al., 1998; Sultan et al., 2001). Moreover, endogenous calpain inhibitors are differentially expressed in slow‐ versus fast‐twitch muscle (Motter et al., 2021). Hence, these fibre type differences may partially be responsible for the post‐RE type I versus IIa isoform fragmentation patterns. Mechanisms aside, it is tempting to speculate how divergent post‐exercise MyHC isotype fragmentation patterns associate with muscle hypertrophy outcomes. In this regard, there is generally a greater increase in type II versus type I fibre cross‐sectional area in response to a variety of resistance training protocols (Fry, 2004; Roberts et al., 2023), and our laboratory has reported this on multiple occasions (Mesquita et al., 2023; Mobley et al., 2017; Ruple et al., 2021; Vann, Osburn et al., 2020). There is also evidence to suggest that plyometric RE leads to significantly more sarcomere damage in type II versus I fibres (∼85% vs. 27%) as assessed by transmission electron microscopy (Macaluso et al., 2012). This prior finding agrees in principle with observations of a more robust post‐RE increase in IIa versus I isoform fragmentation. However, we temper our enthusiasm for various reasons. First, the compact pattern of IIa isoform fragmentation made it more difficult to distinguish between the intact protein and protein fragments (see lane profile in Figure 4b). Additionally, the type I isoform yielded more pre‐exercise immunoreactive fragments across numerous participants compared to the type IIa isoform. Hence, the utilization of more refined approaches (e.g., longer electrophoresis run times for IIa assays) is needed to determine the extent of IIa isoform fragmentation. Future investigations parsing the mechanisms responsible for fibre type‐specific differences in fragmentation and/or if these differences are associated with fibre type‐specific responses to training are also warranted.

The robust MyHC_Total_ fragmentation response following 2 weeks of disuse atrophy in both sexes is another novel finding that is worthy of discussion. Multiple pathways catalyse MPB including calpain‐mediated proteolysis, lysosome‐mediated autophagy, and the ATP‐dependent ubiquitin–proteasome pathway (UPP) (Pasiakos & Carbone, 2014; Tipton et al., 2018). Although human and rodent disuse studies support an upregulation in surrogate skeletal muscle and/or blood markers related to these processes (Doering et al., 2022; Jones et al., 2004; Moller et al., 2019; Talbert et al., 2013; Vazeille et al., 2008; Wall et al., 2014), MPB has been reported to remain unaltered or paradoxically reduced during 4 and 21 days of different disuse models in younger males (Brook et al., 2022; Symons et al., 2009). Indeed, this may be a limitation of tracer methods and assumptions used to assess MPB as posited by O'Reilly et al. (2023). One limitation herein is the lack of time course biopsies during leg immobilization. In this regard, an interesting interrogation would include examining MyHC fragmentation patterns 1, 3 and/or 5 days following leg bracing. Notwithstanding, the current data suggest that the breakdown of MyHC occurs with VL muscle atrophy following disuse in humans, and this simple‐to‐execute immunoblot‐based assay may serve as a viable proteolysis surrogate for future disuse studies.

The robust sample size of the 10weekRT study allowed for comparisons based on training status and sex that warrant further discussion. If indeed post‐RE MyHC fragmentation is a surrogate for myofibre damage, then the data of Figure 6 suggest that a naïve RE bout may elicit a greater 24 h post‐exercise damage response, as the response was attenuated in the trained state. These findings are supported by Damas et al. (2018), who investigated the global transcriptome signature in nine young men. Muscle biopsies were conducted at rest and 24 h post‐resistance training (RT), both before (untrained state) and after (trained state) a 10‐week resistance training program. An upregulation of genes associated with the UPP, the calpain pathway and extracellular matrix (ECM) remodelling were observed 24 h after single RE bouts, with a more notable increase observed in the untrained state. Additionally, these results were accompanied by a reduction in muscle damage (Damas et al., 2016).

Interestingly, the attenuation of increased fragmentation in the trained state was greater for men than for women. This counters the notion that oestradiol confers protection against post‐exercise muscle damage, albeit considerable debate has ensued suggesting that this phenomenon is confined to rodents (Pizza et al., 2009). Moreover, sex‐based differences in anabolic signalling, mRNAs associated with proteolysis, or MPS rates are minimal between young adult men and women over a 24 h post‐exercise period (West et al., 2012). Women tend to experience less acute fatigue (Hunter, 2014), and thus it can be speculated that more disruption per session limits recovery between sessions. However, while some evidence exists showing greater relative strength decrements (Fredsted et al., 2008; Sewright et al., 2008), a heightened post‐exercise inflammatory response (MacIntyre et al., 2000), and a heightened post‐exercise blood creatine kinase response (Fredsted et al., 2008) compared to males, other evidence contradicts these findings (Dannecker et al., 2012; Marshall et al., 2020; Wolf et al., 2012). Mechanisms potentially responsible for this sex‐divergent response were not interrogated. However, the significance of this finding is questionable for a couple of reasons. First, both sexes demonstrated similar hypertrophic outcomes in prior 10weekRT study publications (Chaves et al., 2024; Scarpelli et al., 2024b). Moreover, the 24 h post‐exercise MyHC_Total_ fragmentation response to the last bout of exercise was attenuated in females and not different between sexes. Notwithstanding, the current study provides additional data to support that sex‐based differences in response to RE exist and provide a further impetus to examine this area of muscle biology.

Finally, our in vitro model added additional mechanistic insight, albeit we caution that the model has limitations, namely, the lack of a loading/contraction stimulus. Notwithstanding, our treatment schematic was based on the premise that serum‐free‐medium treatments would best represent our human disuse given the removal of an anabolic stimulus (i.e., serum). Conversely, we hypothesized that high‐serum treatments would induce anabolism. Our data support our hypotheses that serum‐free treatments were catabolic and high‐serum treatments were anabolic. Moreover, calpain inhibition (via calpeptin) reduced MyHC_Total_ fragmentation implicating the role of calpains in this process. However, the lack of MyHC_Total_ fragmentation with serum‐free‐induced myotube atrophy counters our original hypothesis and implies that a reduction in protein synthesis (rather than enhanced proteolysis) is the likely culprit. It is also difficult to explain why calpeptin treatments reduced intact MyHC protein content while paradoxically increasing protein synthesis levels. Indeed, there is an intricate interplay between muscle protein synthesis and muscle proteolysis, and we have published prior evidence suggesting that the inhibition of the calpain and ubiquitin proteasome systems (6 h treatments) significantly reduces protein synthesis in C_2_C_12_ myotubes (Osburn et al., 2021). Hence, a reduced accumulation of intact MyHC may be a consequence of transient calpeptin‐induced reductions in protein synthesis (i.e., within 24 h), and the rebound in protein synthesis at later treatment time points may be compensatory. Aside from these tangential findings, however, it is important to note that the significant insight gained from these experiments is that calpains are likely largely responsible for MyHC protein fragmentation.

### 4.1 Conclusions

In summary, MyHC fragmentation occurs in response to RE bouts and disuse atrophy in humans. A refined fragmentation response with 10 weeks of resistance training and more refined responses in well‐trained participants suggest this an adaptive process. Importantly, we posit that our easy‐to‐execute immunoblot‐based technique has promising utility with RE or disuse studies. More research is needed to determine how different exercise modalities (e.g., concurrent training), ageing or certain diseases that promote skeletal muscle atrophy (e.g., hyper‐metabolic stress, cancer‐cachexia) affect MyHC fragmentation. Research into the physiological consequences of different fragmentation responses as well as the fate of MyHC fragments is also warranted.

## AUTHOR CONTRIBUTIONS

Daniel L. Plotkin and Michael D. Roberts: conceptualization, formal analysis, and writing of primary draft; Daniel L. Plotkin, Madison L. Mattingly, Derick A. Anglin, J. Max Michel, Michael D. Roberts: experimentation; Joshua S. Godwin, Mason C. McIntosh, João G. A. Bergamasco, Nicholas J. Konto, Maíra C. Scarpelli, Vitor Angleri, Lemuel W. Taylor, Darryn S. Willoughby, C. Brooks Mobley, Andreas N. Kavazis, Carlos Ugrinowitsch, Cleiton A. Libardi: collaborative insight throughout experiments and/or significant resources devoted to project outcomes; all‐co‐authors: manuscript editing and approval of final submission. All authors have read and approved the final version of this manuscript and agree to be accountable for all aspects of the work in ensuring that questions related to the accuracy or integrity of any part of the work are appropriately investigated and resolved. All persons designated as authors qualify for authorship, and all those who qualify for authorship are listed.

## CONFLICT OF INTEREST

None of the authors disclose conflicts of interest related to these data.

## Data Availability

All individual respondent data can be visualized in figures. Raw data will be made available by the corresponding author (mdr0024@auburn.edu) upon reasonable request.
